# The Effect of Milling Time on the Microstructural Characteristics and Strengthening Mechanisms of NiMo-SiC Alloys Prepared via Powder Metallurgy

**DOI:** 10.3390/ma10040389

**Published:** 2017-04-06

**Authors:** Chao Yang, Ondrej Muránsky, Hanliang Zhu, Gordon J. Thorogood, Maxim Avdeev, Hefei Huang, Xingtai Zhou

**Affiliations:** 1 Shanghai Institute of Applied Physics, Chinese Academy of Sciences (CAS), Shanghai 201800, China; yangchao@sinap.ac.cn (C.Y.); zhouxingtai@sinap.ac.cn (X.Z.); 2 University of Chinese Academy of Sciences, Beijing 100049, China; 3 Australian Nuclear Science and Technology Organisation, Lucas Heights, NSW 2234, Australia; hgz@ansto.gov.au (H.Z.); gjt@ansto.gov.au (G.J.T.); max@ansto.gov.au (M.A.); 4 School of Materials Science and Engineering, University of New South Wales, Sydney, NSW 2052, Australia

**Keywords:** powder metallurgy, DPS strengthening, spark plasma sintering, transmission electron microscopy, electron backscatter diffraction, molten salt reactor

## Abstract

A new generation of alloys, which rely on a combination of various strengthening mechanisms, has been developed for application in molten salt nuclear reactors. In the current study, a battery of dispersion and precipitation-strengthened (DPS) NiMo-based alloys containing varying amounts of SiC (0.5–2.5 wt %) were prepared from Ni-Mo-SiC powder mixture via a mechanical alloying (MA) route followed by spark plasma sintering (SPS) and rapid cooling. Neutron Powder Diffraction (NPD), Electron Back Scattering Diffraction (EBSD), and Transmission Electron Microscopy (TEM) were employed in the characterization of the microstructural properties of these in-house prepared NiMo-SiC DPS alloys. The study showed that uniformly-dispersed SiC particles provide dispersion strengthening, the precipitation of nano-scale Ni_3_Si particles provides precipitation strengthening, and the solid-solution of Mo in the Ni matrix provides solid-solution strengthening. It was further shown that the milling time has significant effects on the microstructural characteristics of these alloys. Increased milling time seems to limit the grain growth of the NiMo matrix by producing well-dispersed Mo_2_C particles during sintering. The amount of grain boundaries greatly increases the Hall–Petch strengthening, resulting in significantly higher strength in the case of 48-h-milled NiMo-SiC DPS alloys compared with the 8-h-milled alloys. However, it was also shown that the total elongation is considerably reduced in the 48-h-milled NiMo-SiC DPS alloy due to high porosity. The porosity is a result of cold welding of the powder mixture during the extended milling process.

## 1. Introduction

Molten Salt Reactors (MSRs) have been selected as a candidate for Generation IV nuclear reactors, due to their inherent safety, economical efficiency, and online refuelling capabilities, as well as their capacity to produce hydrogen from seawater using the waste heat of the reactor [1,2]. However, the structural materials in the primary molten salt loop will be subjected to extreme operating environments, such as high temperatures, strong neutron irradiation and severe corrosion from molten salt coolants [3]. Hastelloy-N, developed at Oak Ridge National Laboratory (ORNL), is generally accepted as the most promising candidate material in many MSR designs due to its superior corrosion resistance in molten salt at high temperatures [4]. Unfortunately, its disadvantages in high-temperature strength and irradiation resistance limit the development of fully commercial MSRs [5].

In our initial study [6,7], a SiC dispersion-strengthened Ni-matrix (Ni-SiC) composite was successfully prepared via a powder metallurgy (PM) process. SiC particles, which possess outstanding thermal stability at temperatures up to 850 °C, were homogeneously dispersed in the Ni matrix [8]. However, the strength of Ni-SiC composite was found not to be satisfactory for application in MSRs [9,10]. Therefore, in the next stage of our materials development we developed a new generation of alloys, which rely on a combination of various strengthening mechanisms. These NiMo-based alloys retain the advantages of SiC dispersion strengthening in addition to precipitation strengthening by nano-scale Ni_3_Si precipitates. Hence, we refer to these novel NiMo-SiC alloys as dispersion- and precipitation-strengthened (DPS) alloys. In addition to the dispersion- and precipitation-strengthening mechanisms, Mo dissolved in a Ni matrix forms a solid-solution, which provides further strengthening of the matrix. As a result of these strengthening mechanisms, NiMo-SiC alloys possess superior mechanical properties compared to simple Ni-SiC composites developed via a similar PM processing route [6,7].

The initial findings of our research on 8-h-milled NiMo-SiC DPS alloys has been published in [11]. However, it has been indicated that 8-h-milled NiMo-SiC DPS alloys show insignificant Hall–Petch (grain-size) strengthening of the NiMo matrix due to agglomeration of Mo_2_C. In order to improve the Hall–Petch strengthening process, it has been suggested that longer milling times might prevent the agglomeration of Mo_2_C, which can then effectively inhibit the NiMo matrix grain growth [11]. Therefore, we prepared a battery of DPS NiMo-SiC alloys containing 0.5, 2.0, and 2.5 wt % of SiC, which were milled for 48 h rather than for 8 h. The present paper then compares the mechanical properties and microstructural characteristics of 48-h-milled NiMo-SiC DPS alloys with 8-h-milled NiMo-SiC DPS alloys [11].

## 2. Methods

The initial powder mixtures containing 16 wt % of Mo powder (99.6% purity, 3.5 μm, bcc), 0.5; 2; 2.5 wt % of SiC (99.9% purity, 30 nm, fcc), and balance of Ni powder (99.6% purity, 3.5 μm, fcc) were prepared by a 48-h high-energy milling process. The high-energy milling was carried out in an agate jar (1 L) with spherically shaped agate balls (Φ 6 mm and Φ 10 mm) via a QM-3SP4 planetary ball mill. The ball-to-powder (BPR) weight ratio is 10:1. The rotation speed of the mill table (disk) was set to 150 revolutions a minute, while four jars sitting on the mill table were rotating on their axes at a speed of 300 revolutions a minute. An additional powder mixture with 16 wt % of Mo and balance of Ni powder was firstly milled for 48 h in order to create a protection coating on the surface of the agate milling balls and jars. The leftover of this powder mixture was discarded before milling the actual NiMo-SiC powder mixture, thus significantly reducing the impurities from milling balls and jars during mechanical alloying.

These initial powder mixtures were then consolidated into a bulk via a spark plasma sintering (SPS) furnace (KCE-FCT-HP D 25/4-SD, Germany) forming NiMo-SiC DPS alloys. A cylindrical graphite die with an inner diameter of 50 mm and a chamber vacuum of 15 mbar were used during the sintering process. The mixture was pulse-heated from 25 to 1100 °C with a heating rate of 75 °C/min and a uniaxial mechanical pressure through graphite punch (50 MPa). After holding at the desired temperature (1100 °C) and pressure (50 MPa) for 10 min, samples were rapidly cooled to room temperature with a cooling rate of 110 °C/min via an indirect water cooling system of pressing graphite punch, while the pressure was released from 50 to 0 MPa.

High-resolution neutron powder diffraction (NPD) measurements were performed at room temperature using the Echidna instrument at the OPAL reactor. Data were obtained using neutrons of wavelength λ = 1.622 Å over a range of 39° to 156° [12]. The Electron Back-Scatter Diffraction (EBSD, Zeiss Auriga 60, 20 kV) and Transmission Electron Microscopy (TEM, JEM-2200FS, 200 kV) were employed to identify minor phases as well as their crystal structure, size, distribution, and interaction with the dislocation sub-structure. The tensile mechanical properties of bulk NiMo-SiC DPS alloys milled for 48 h were obtained at room temperature using an Instron tensile machine with a strain rate of 0.06 mm/min.

## 3. Results and Discussion

### 3.1. Mechanical Properties

**Figure 1:**
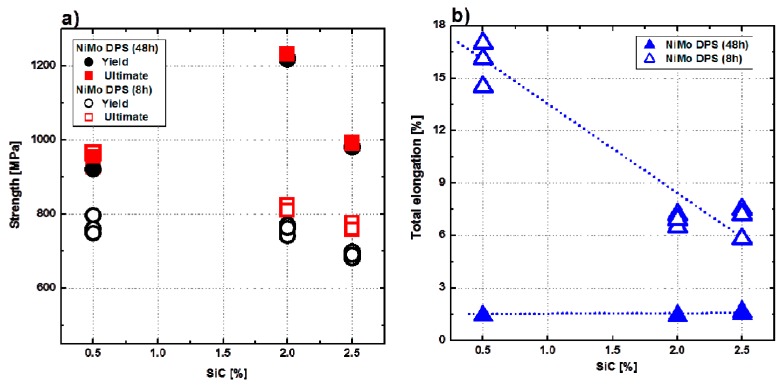
Variations of (**a**) yield and tensile strength; and (**b**) total elongation of 48-h-milled and 8-h-milled NiMo-SiC dispersion and precipitation-strengthened (DPS) alloys with an increasing amount of SiC in the initial powder mixture.

Figure 1 presents the yield strength (YS), ultimate tensile strength (UTS), and total elongation (L) of 48-h-milled NiMo-SiC DPS alloys together with the results from our initial study concerning the 8-h-milled NiMo-SiC DPS alloys [11]. Both YS and UTS of 48-h-milled NiMo-SiC DPS alloys were found to be consistently higher compared to the 8-h-milled NiMo-SiC DPS alloys, while showing significantly lower total elongation (L). Regarding the total elongation shown in Figure 1b, it becomes clear that the ductility is significantly reduced in the 48-h-milled compared to the 8-h-milled NiMo-SiC DPS alloys. Interestingly, the total elongation of 48-h-milled NiMo-SiC DPS alloys is more-or-less constant regardless of SiC content (solid symbol in Figure 1b), while the total elongation is strongly dependent on the SiC content in the case of 8-h-milled NiMo-SiC DPS alloys (open symbol in Figure 1b) [11]. This suggests that the SiC content has only secondary effect on the ductility of 48-h-milled NiMo-SiC DPS alloys.

**Figure 2:**
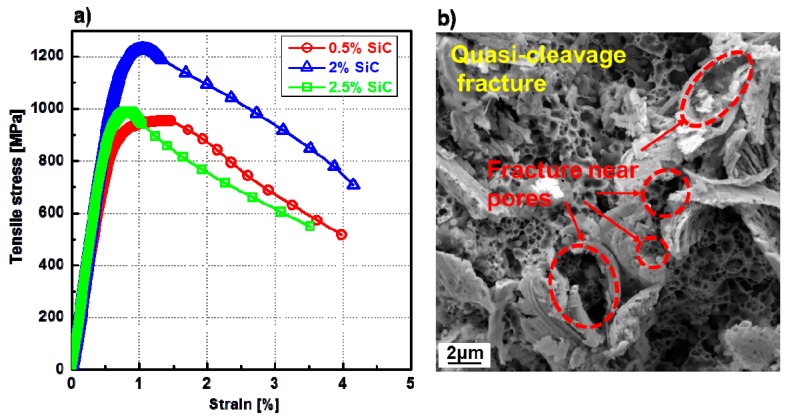
(**a**) Stress-strain curves of 48-h-milled NiMo-SiC DPS alloys with an increasing amount of SiC in the initial powder mixture, and (**b**) SEM image of relative fracture tomography (shown at 10 nm resolution).

Figure 2 shows the stress-strain curves (Figure 2a) of the 48-h-milled NiMo-SiC DPS alloys and fracture surface (Figure 2b) obtained using a scanning electron microscope (SEM, Zeiss Ultra-Plus). It is clear that all the 48-h-milled NiMo-SiC DPS alloys fractured at the very beginning of the plastic region. A number of dimples and cleavage planes were found on the fracture surface as can be seen in Figure 2b. The fracture of 48-h-milled NiMo DPS alloys demonstrated a typical quasi-cleavage fracture characteristic. Thus, it seems that the lower-than-expected total elongation is a result of high porosity in 48-h-milled NiMo-SiC DPS alloys.

### 3.2. Density Analysis

**Figure 3:**
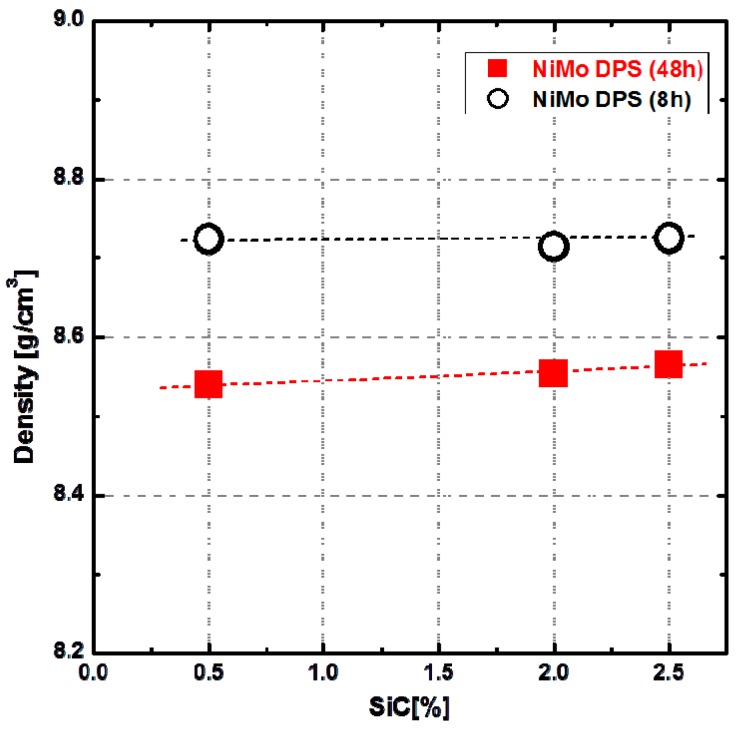
Density of 48-h-milled and 8-h-milled NiMo-SiC DPS alloys with an increasing amount of SiC in the initial powder mixture.

The density of the 8-h-milled and 48-h-milled NiMo-SiC DPS alloys was measured by Archimedes method, and the results are shown in Figure 3. It is clear that the density of the 48-h-milled samples is considerably lower than that of 8-h-milled samples. This indicates that the high porosity is indeed the main reason for unexpectedly low ductility of 48-h-milled NiMo-SiC DPS alloys. This further shows that the milling time has direct effect on the alloy density. In order to investigate the reasons behind this correlation between the milling time and the density of the final alloy, we examined milled powder mixtures in more detail.

**Figure 4:**
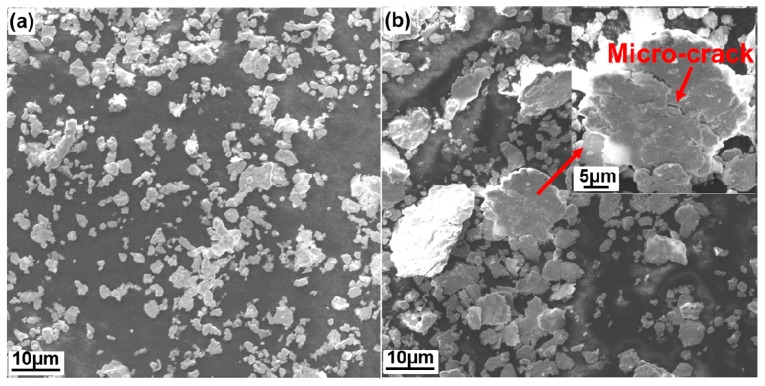
SEM images of powder mixture milled for (**a**) 8 h and (**b**) 48 h with 0.5 wt % of SiC in the initial powder mixture (shown at 10 nm resolution).

Morphology of the powder mixture milled for 8 h and 48 h was obtained using SEM (Zeiss Ultra-plus, 15 KV) and it is shown in Figure 4. It becomes apparent that the powder particles in the powder mixture milled for 48 h are much larger in size compared to the powder particles found in the power mixture milled for 8 h. It is believed that this is due to so-called cold welding, which takes place during the milling process [8]. The cold-welding is expected to be more pronounced in the alloys milled for 48 h. In addition, numerous micro-cracks can be seen in the large powder particles, as is evident in Figure 4b. The presence of micro-cracks is important, because vacuum condition in the initial stage of sintering can effectively expel air/gas between two separated powder particles, but residual air/gas can be enclosed into the micro-cracks of powder particles due to the plastic deformation from discharge effect and compaction in the second stage of sintering. Once a pore is formed in this stage, it will not be removed in the following sintering stages [13,14]. Thus, it is believed that the larger particle size and the existence of micro-cracks found in the powder mixture milled for 48 h result in the poor density of 48-h-milled NiMo-SiC DPS alloys.

### 3.3. Microstructure Characterization

Although high porosity significantly reduced the total elongation of the 48-h-milled NiMo-SiC DPS alloys, the tensile strength (YS and UTS) of these alloys is clearly higher than alloys milled for 8 h, which is shown in Figure 1. To investigate possible reasons for this obvious improvement in strength, a rigorous microstructure characterization has been conducted. A number of constituent phases (carbides, silicides) presenting in the microstructure of the 48-h-milled NiMo-SiC DPS alloys have been identified using a range of diffraction techniques available at the Australian Nuclear Science and Technology Organization (ANSTO); see methods section.

#### 3.3.1. Neutron Diffraction Analysis

**Figure 5:**
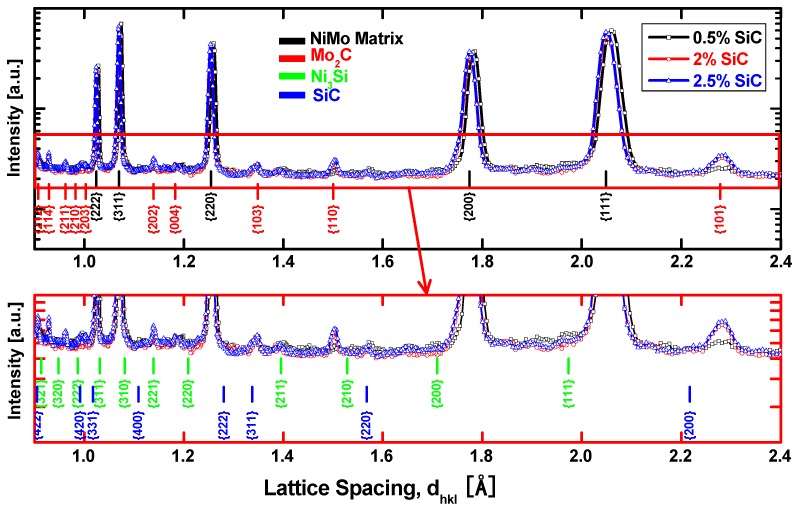
Neutron diffractograms of 48-h-milled NiMo-SiC DPS alloys with an increasing amount of SiC in the initial powder mixture.

Figure 5 presents the comparison of full neutron diffraction patterns of the 48-h-milled NiMo-SiC DPS alloys. The solid solution of Mo in Ni is formed during sintering, while due to the high affinity of Mo and C a reaction between SiC and Mo leads to the formation of Hexagonal Closed-Packed (HCP) Mo_2_C [11]. In addition, SiC and Ni_3_Si peaks were also detected via neutron diffraction. As one would expect, the Mo_2_C diffraction peaks increase with an increase of SiC content in the initial powder mixture. It is further interesting to see the shift of the NiMo diffraction peaks, which suggests a variation in the crystal lattice parameter of the NiMo matrix [11].

**Figure 6:**
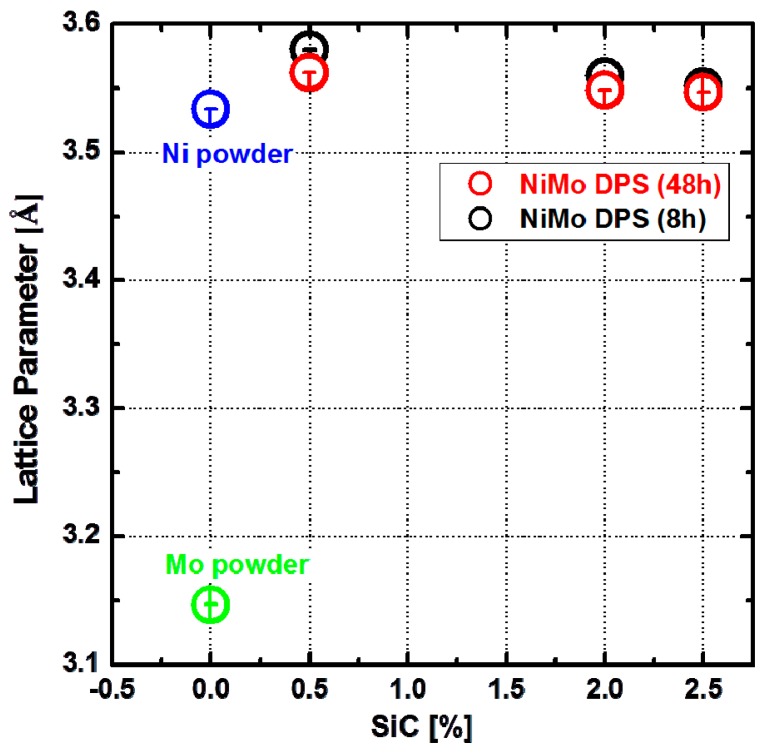
Lattice parameters of Ni and Mo in as-milled condition, as well as Ni-Mo matrix in bulk 48-h-milled and 8-h-milled NiMo-SiC DPS alloys with an increasing amount of SiC in the initial powder mixture.

In order to evaluate the lattice parameter of the NiMo matrix, a full diffraction pattern analysis (LeBail) was undertaken using the GSAS software package [15,16,17]. Figure 6 presents variations of the lattice parameter of the NiMo matrix as a function of SiC content in the initial powder mixture. The lattice parameters of pure Ni and Mo powders in the as-milled condition are shown for comparison. As one would expect when a NiMo solid-solution is formed during sintering, the Ni lattice is stretched by the presence of Mo atoms, forming a single-phase solid-solution of NiMo. It is, however, interesting to see that the lattice parameter of the NiMo matrix decreased with increasing SiC content, approaching that of pure Ni powder. This suggests a lower content of Mo in solid-solution of the NiMo matrix as the content of SiC increased. It is expected that this will have an effect on the solid-solution strengthening mechanism. In addition, it can be seen in Figure 6 that the lattice parameter of NiMo matrix in the 48-h-milled NiMo-SiC DPS alloys is consistently lower than that in 8-h-milled alloys. This is a somewhat unexpected result because one could assume that the longer milling time will lead to a better mechanical alloying and thus higher content of Mo in the Ni matrix. However, it seems that the longer time promoted the reaction between Mo and SiC, which ultimately led to a lower amount of Mo in the Ni matrix and slightly higher Mo_2_C content. It is believed the reaction between Mo and SiC is promoted by more uniform distribution of Mo and SiC in the powder mixture after milling for a longer time (48 h).

#### 3.3.2. EBSD Analysis

**Figure 7:**
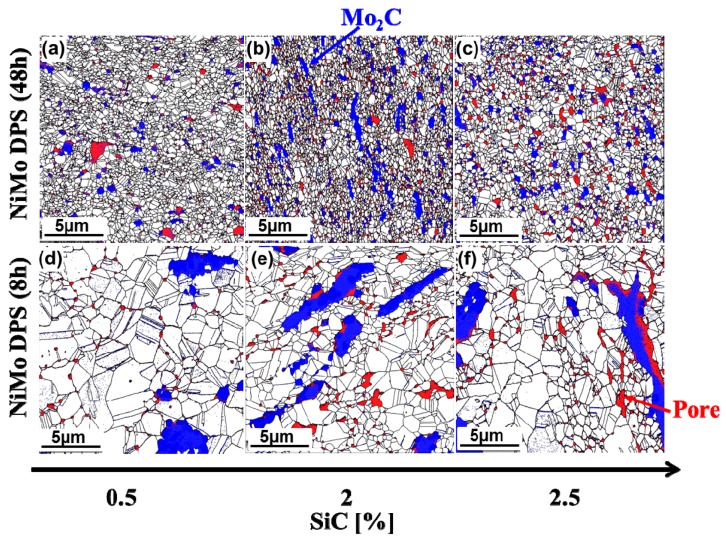
Electron Back Scattering Diffraction (EBSD) plots of Mo_2_C distribution in 48-h-milled NiMo-SiC DPS alloys with the initial SiC content of (**a**) 0.5, (**b**) 2 and (**c**) 2.5 wt %, and 8-h-milled NiMo-SiC DPS alloys with the initial SiC contents of (**d**) 0.5, (**e**) 2 and (**f**) 2.5 wt %. Blue areas show Mo_2_C distribution and red areas show pores, within the matrix (white areas).

In our initial study [11], it was found that Mo_2_C can inhibit the grain growth of the NiMo matrix during sintering, leading to an increase in Hall–Petch (grain-size) strengthening of the alloy. However, the large agglomerates of Mo_2_C in the 8-h-milled NiMo-SiC DPS alloys largely limited this function. It was suggested in [11] that longer milling time might improve the distribution of Mo_2_C, thus refining grain size of the NiMo matrix. EBSD analysis was conducted in this study to investigate the microstructural characteristics of the Mo_2_C distribution and grain-size variations in the matrix. Figure 7 presents EBSD maps of the 48-h-milled and 8-h-milled NiMo-SiC DPS alloys. Grain as well as twin boundaries of NiMo matrix are shown in black, while pores in NiMo matrix and pixels indexed as HCP Mo_2_C are shown in red and blue, respectively. The EBSD maps clearly show the Mo_2_C distribution in the matrix of the 48-h-milled NiMo-SiC DPS alloys is more uniform compared with the 8-h-milled alloys. Also, the size of Mo_2_C agglomerates in the 48-h-milled sample was much smaller than in 8-h-milled NiMo-SiC DPS alloys. In addition, one can see numerous pores randomly dispersed in the matrix of all prepared NiMo-SiC DPS alloys. Note that due to the large magnification in EBSD images, we cannot show the entire volume fraction of pores in the matrix, thus limiting a full comparison with the density results above (Section 3.2).

**Figure 8:**
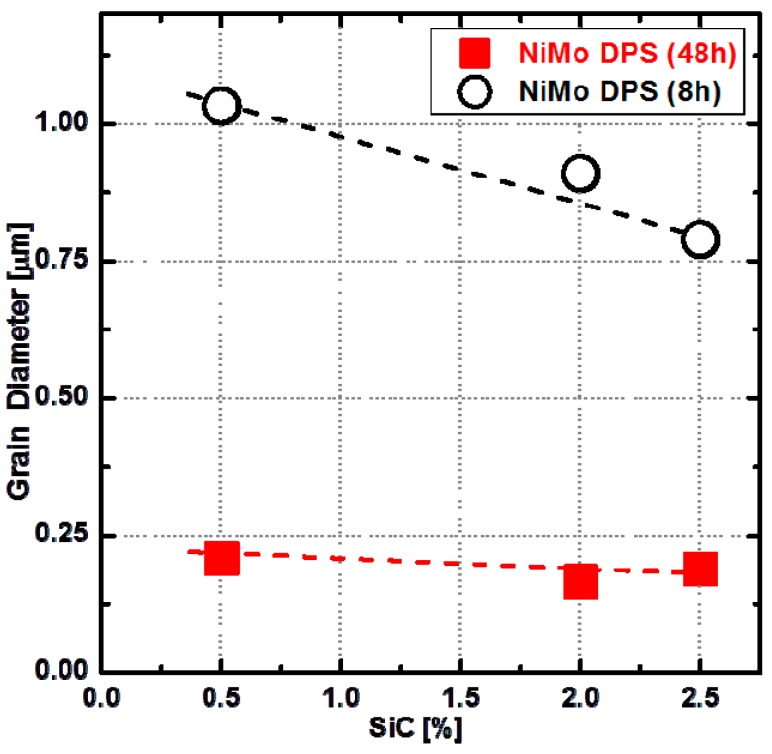
Variations of average grain diameters in 48-h-milled and 8-h-milled NiMo-SiC DPS alloys with SiC contents from 0.5 to 2.5 wt % in the initial powder mixture.

In order to evaluate the grain size of the NiMo matrix, a detailed analysis of EBSD maps was performed employing the MTEX [18,19,20] toolbox for Matlab. These results are shown in Figure 8 as a function of SiC content in the initial powder mixture and milling time. The test shows that the grain size of the NiMo matrix of the 48-h-milled NiMo-SiC DPS alloys is significantly smaller than that of the 8-h-milled alloys. This confirms the initial assumption made in [11] that an increased milling time leads to a more uniform distribution of Mo_2_C, which then leads to a finer grain size by inhibiting the grain growth during sintering. It is further interesting to see that the grain size of NiMo matrix is independent of the variation of SiC content in the case of 48-h-milled NiMo-SiC DPS alloys. Based on the NPD results in Section 3.3.1, an increase of SiC content in the initial powder mixture can result in a significant increasing amount of Mo_2_C in the NiMo matrix of bulk samples. This clearly suggests that there is no significant effect of Mo_2_C content on the variation of grain size in 48-h-milled alloys. Thus, the inhibition of grain growth is dominated by the uniform distribution of Mo_2_C, rather than its content in the material.

#### 3.3.3. TEM Analysis

**Figure 9:**
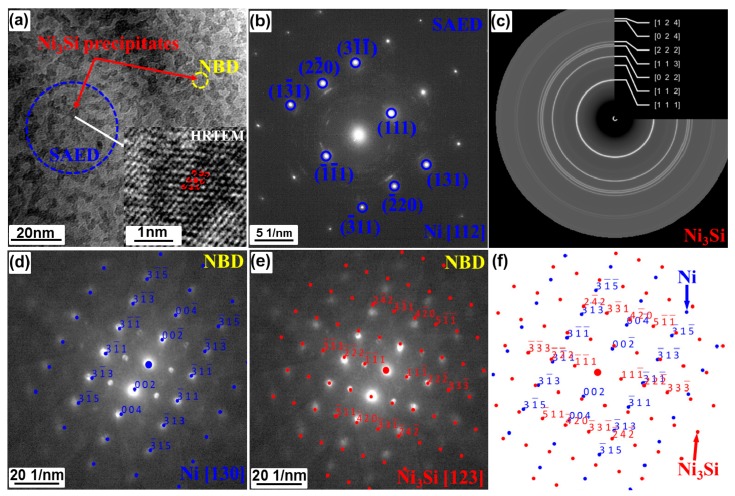
(**a**) Bright field and high resolution TEM images; (**b**,**c**) selected area electron diffraction (SAED); (**d**,**e**) nano-diffraction (NBD) and (**f**) simulative patterns of Ni_3_Si and Ni in 48-h-milled NiMo-2.5% SiC DPS alloys.

TEM technique was used to identify the characteristics and presence of minor phases in the prepared NiMo-SiC DPS alloys. It has been found that all the alloys have similar features regardless of the amount of SiC in the initial powder mixture and the milling time. Hence, here we present the detail analysis of the 48-h-milled NiMo-2.5% SiC DPS specimen. The same TEM analysis was used on the other alloy specimens. A bright field (BF) TEM image with a high-resolution (HR) TEM image, together with selected area electron diffraction (SAED) and nano-diffraction (NBD) patterns, was recorded. These results are shown in Figure 9. It is clear from the BFTEM image (Figure 9a) that expected Ni_3_Si nano-precipitates [11] are not homogeneously distributed within the matrix, but rather agglomerate in small clusters about 5 nm in size. The HRTEM (inset in Figure 9a) of one of these clusters suggests that Ni_3_Si nano-precipitates have face-cantered cubic crystal structure and their size is of about 1–3 nm. In order to confirm the crystal structure of these nano-precipitates, the SAED pattern has been obtained in Figure 9b; it contains both the diffraction rings and spots. The strong SAED spots coincide well with those of the Ni matrix in the zone axis of [112]. The SAED rings are revealed and identified using CrysTBox software [21], as shown in Figure 9c; they are found to be close to those for the Ni_3_Si phase. In order to further confirm the crystal structure of the nano-precipitates, the NBD patterns were obtained (Figure 9d,e) from another cluster of Ni_3_Si nano-precipitates (Figure 9a). Because the nano-precipitates are small, the NBD patterns contain the diffraction spots from both the NiMo matrix and Ni_3_Si nano-precipitate. The zone axis for the Ni matrix is identified as [130] (Figure 9d), while the spots for the nano-precipitate are close to the simulation patterns of Ni_3_Si in the zone axis of [123] (Figure 9e). The simulation patterns for Ni and Ni_3_Si were performed using Singlecrystal™ software (CrystalMaker Software Limited, Oxfordshire, UK) [22,23]. Figure 9f presents the simulation of the combined NBD patterns for NiMo matrix and Ni_3_Si nano-particle in the zone axis of [130] and [132] respectively. It seems that there is no clear relationship between the Ni_3_Si and the matrix, as the Ni_3_Si nano-precipitates seem to randomly precipitate from the Ni matrix.

**Figure 10:**
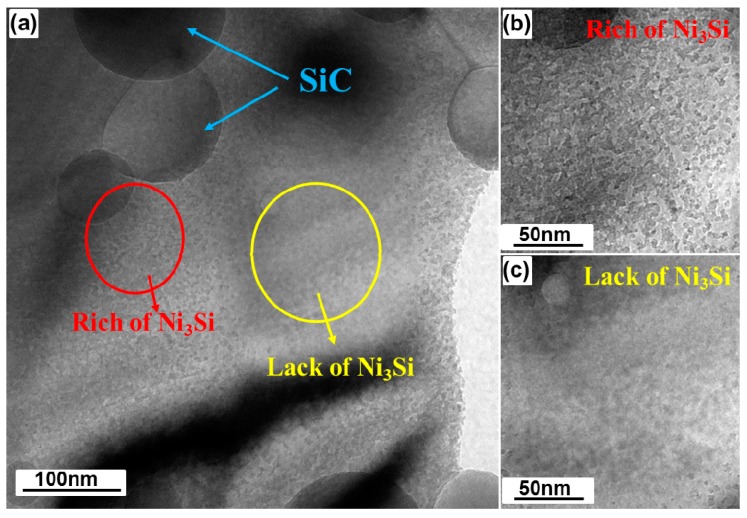
Bright field TEM images of (**a**) SiC and Ni_3_Si distribution, the areas (**b**) rich and (**c**) lack of Ni_3_Si in the matrix of 48-h-milled NiMo-2.5% SiC DPS alloys.

As shown in Figure 9a, the Ni_3_Si nano-particles are unevenly distributed within the matrix. Figure 10 sheds light on this uneven distribution of the Ni_3_Si nano-precipitates. It becomes evident from Figure 10 that the areas near the SiC particles are rich in the nano-precipitates (Figure 10b), whereas the regions far from the SiC particles lack the Ni_3_Si nano-precipitates (Figure 10c). The reason for this is simply that there is a higher concentration of disassociated Si atoms in the vicinity of SiC particles. Hence, there is a higher amount of Ni_3_Si nano-precipitates near the SiC/matrix interfaces, while there are no Ni_3_Si nano-precipitates present in the region free of SiC particles.

**Figure 11:**
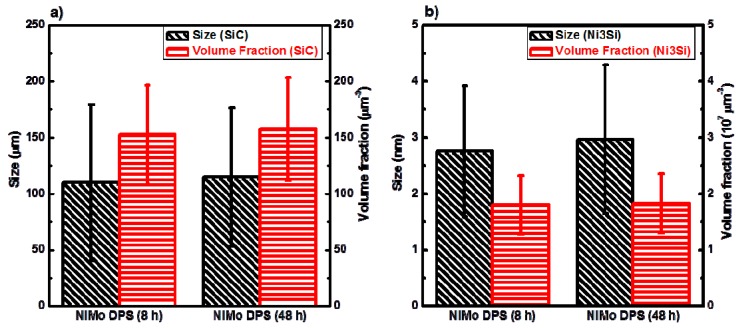
Size distribution and volume fraction of (**a**) SiC particles and (**b**) Ni_3_Si nano-precipitates in the 8-h-milled and 48-h-milled NiMo-2.5% SiC DPS alloys.

The combination of SiC particles and Ni_3_Si nano-particles can effectively inhibit dislocation motions in NiMo-SiC DPS alloys, resulting in an significant increase of mechanical strength [11]. Because both types of strengthening mechanisms – dispersion- and precipitation-strengthening—are directly correlated to the size and volume fraction of particles, the effect of milling time on the variation of the size and volume fraction of SiC particles and Ni_3_Si nano-precipitates has been studied. In order to minimize statistical errors, 10 of the BRTEM images captured from randomly-selected areas in each condition were used for quantitative analysis of present SiC particles and Ni_3_Si nano-precipitates. Figure 11 presents the average size and volume fraction of SiC particles and Ni_3_Si nano-precipitates in the 8-h-milled and 48-h-milled samples. It is found that the difference in the size and volume fraction of both types of particles between the 8-h-milled and 48-h-milled alloys is within the standard deviation of the measurements. This suggests that the milling time has no significant effect on the size and distribution of SiC particles and Ni_3_Si nano-precipitates. Hence, the difference observed in the mechanical properties is more likely linked to the volume fraction and distribution of Mo_2_C, and the grain size of NiMo matrix (Hall–Petch strengthening).

**Figure 12:**
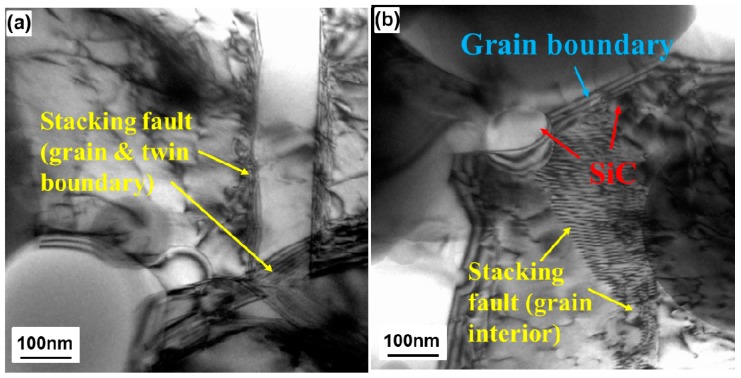
Bright-field TEM images of dislocation motions inhibited by (**a**) grain & twin boundary and (**b**) SiC, Ni_3_Si and grain boundary in the tensile tested 48-h-milled NiMo-0.5% SiC DPS alloy.

Figure 12 presents BFTEM images showing the interactions between dislocations and other microstructural features in the tensile-tested 48-h-milled NiMo-0.5% SiC DPS alloy. It is clear that dislocation motion is effectively inhibited by the presence of the grain and twin boundaries. The inhibited dislocations can transform into stacking faults near the grain and twin boundaries, as shown in Figure 12a. This indicates that grain boundaries also play an important role in strengthening the alloy by Hall–Petch strengthening. In addition, the dislocation motion is further inhibited in the vicinity of SiC particles, Ni_3_Si nano-precipitates and grain boundaries (Figure 12b). These inhibited dislocations also transform into stacking faults in the grain interiors, thus strengthening the NiMo matrix. This suggests that Hall–Petch strengthening provides a significant contribution to a higher strength of the 48-h-milled NiMo-SiC DSP alloys (Figure 1) compared to 8-h-milled NiMo-SiC DSP alloys.

## 4. Conclusions

The effect of milling time on the microstructural and mechanical properties of the developed DPS NiMo-based alloys was investigated. In comparison with a milling time of 8 h, the long milling time of 48 h limits the grain growth of the matrix by producing well-dispersed Mo_2_C particles during the sintering process. Hence, the longer milling time results in a much finer grain size in the 48-h-milled NiMo-SiC DPS alloys compared to the 8-h-milled ones. The same as in the 8-h-milled alloy, the solid solution of Mo in the Ni matrix of 48-h-milled NiMo DPS alloys provides solid-solution strengthening, the uniform dispersion of SiC particles provides dispersion strengthening, while the precipitation of nano-scale Ni_3_Si particles provides precipitation strengthening. Moreover, larger amounts of grain boundaries greatly increase the Hall–Petch strengthening, resulting in a much better strength of the 48-h-milled NiMo-SiC DPS alloys compared with the 8-h-milled NiMo-SiC DPS alloys. Note that 2 wt % of SiC in the initial powder mixture is a balanced content, which leads to the most significant synthetic effect of strengthening on the materials. However, the existence of micro-cracks caused by cold welding during milling results in the poor density of 48-h-milled NiMo-SiC DPS alloys. The poor density leads to the unexcitedly low total elongation of these alloys. Therefore, it is suggested that forging or hot rolling, as well as heat treatment, need to be used in the future preparation to obtain NiMo-SiC DPS alloys with higher density and better ductility.
